# Feasibility of a rapid response mechanism to meet policymakers' urgent needs for research evidence about health systems in a low income country: a case study

**DOI:** 10.1186/s13012-014-0114-z

**Published:** 2014-09-10

**Authors:** Rhona M Mijumbi, Andrew D Oxman, Ulysses Panisset, Nelson K Sewankambo

**Affiliations:** College of Health Sciences, Makerere University, Kampala, Uganda; Norwegian Knowledge Center for the Health Services, St Olavs plass, Oslo, 0130 Norway; World Health Organization, Avenue Appia 20, Geneva 27, 1211 Switzerland

**Keywords:** Knowledge translation, Evidence-informed policy, Health systems research, Health policy, Barriers for evidence-based policies, Rapid response mechanisms, Uganda, Low and middle income countries

## Abstract

**Objectives:**

Despite the recognition of the importance of evidence-informed health policy and practice, there are still barriers to translating research findings into policy and practice. The present study aimed to establish the feasibility of a rapid response mechanism, a knowledge translation strategy designed to meet policymakers' urgent needs for evidence about health systems in a low income country, Uganda. Rapid response mechanisms aim to address the barriers of timeliness and relevance of evidence at the time it is needed.

**Methods:**

A rapid response mechanism (service) designed a priori was offered to policymakers in the health sector in Uganda. In the form of a case study, data were collected about the profile of users of the service, the kinds of requests for evidence, changes in answers, and courses of action influenced by the mechanism and their satisfaction with responses and the mechanism in general.

**Results:**

We found that in the first 28 months, the service received 65 requests for evidence from 30 policymakers and stakeholders, the majority of whom were from the Ministry of Health. The most common requests for evidence were about governance and organization of health systems. It was noted that regular contact between the policymakers and the researchers at the response service was an important factor in response to, and uptake of the service. The service seemed to increase confidence for policymakers involved in the policymaking process.

**Conclusion:**

Rapid response mechanisms designed to meet policymakers' urgent needs for research evidence about health systems are feasible and acceptable to policymakers in low income countries.

**Electronic supplementary material:**

The online version of this article (doi:10.1186/s13012-014-0114-z) contains supplementary material, which is available to authorized users.

## Background

A large amount of health research done globally to help improve lives and strengthen health systems results in about one million publications annually and another equal amount of unpublished work [1]-[3]. However, many of the findings from this research are not used optimally, and this represents missed opportunities for improved patient care and management, resource allocation and strengthened health systems. These missed opportunities and the need to correct them and their consequences have been recognized. While meeting in Mexico a decade ago and in Bamako four years later, health ministers from United Nations member states noted that if existing interventions were adequately adopted, health systems would be stronger and better positioned to deal with current global health challenges [4]. They noted that research had a crucial but under-recognized role in strengthening health systems, and improving the equitable distribution of services. They called for member states to promote knowledge translation (KT) and exchange through evidence-informed policies and policy-informed research, among other things [5].

The use of evidence in decision making is a vital element in improving healthcare delivery, and therefore health system outcomes, and in reducing the waste of resources [6],[7]. Evidence may be used to inform background discussions about the subject at hand; it may aid the definition of the problem, identify the different policy or practice options to consider, or inform implementation strategies in terms of barriers and facilitators [8]. It may also help identify new subjects for the policy agenda and evaluate the impacts of policies too [9].

Both researchers and policymakers acknowledge the importance of evidence-informed policy or decision making and practice [10],[11]. However they have quickly learned that research results rarely passively get adopted into policy and practice [12],[13], resulting in a persistent `know-do' gap - the difference between what we know from research and other sources of evidence, and what we actually do in practice or incorporate in our policies [14]. When health systems fail to use evidence optimally, the result is inefficient use of the available resources and subsequently a reduction in both quantity and quality of healthcare and health outcomes [9],[15]. In fact, the inability for health systems in many low and middle income countries to effectively use evidence to inform their decisions and interventions is cited as a major obstacle to the achievement of the Millennium Development Goals [16],[17].

Efforts to promote the uptake of research evidence in policy making are the subject of several KT strategies, which aim to systematically and transparently provide access to, appraise and contextualize evidence as an input into the policymaking process [18]. Such efforts have been intensified globally including in low and middle income countries. In the African, Asian, and Latin American regions, several KT platforms, including EVIPNet and partners, are developing and evaluating KT strategies. The Ugandan country node of the Regional East African Community Health policy initiative (REACH-PI) based at Makerere University is one of these EVIPNet partners, who in collaboration with the Supporting Use of Research Evidence (SURE) project, is carrying out research on different KT strategies [19]. Others include the KT Network Africa [20], the Cochrane collaboration [21], Evidence for Health Policy in Vietnam (VINE) [22], the African Institute for Development Policy (AFIDEP) [23], Malawi's Knowledge Translation Platform, KTPMalawi [24], and the Evidence to Policy (E2P) in Argentina, Bangladesh and Nigeria [25].

These platforms have contributed to the evolving understanding of factors that affect the `know-do' gap. Several factors have been consistently cited as affecting the uptake of evidence during decision making processes; personal contact between researchers and decision makers, timely relevance of the research evidence in line with the decision making process, and summaries with policy recommendations were common facilitators of KT [26]-[28]. Other factors affecting the process included mutual trust (in this case mistrust) between research producers and policymakers, and decision makers' power and budgetary factors. Another significant factor is that policy and decision making happen at several different levels of governance. It has been noted that evidence may be used more at higher levels than lower ones [29]. Kasonde and Campbell [30] emphasize how knowledge translation is context specific. Decisions are made under different contexts, and one of these is time. Although policy is rarely made in a short period, policy decisions and positions are in fact often made rapidly - often within hours or days [31]. There may also arise urgent situations - say out of parliament, the media, and from real or perceived crises - in which a decision needs to be made in a short time. For a policymaker in this situation, the timeliness and relevance of research evidence if it is to be used to support the process would be a major facilitating or hindering factor. A framework developed to assess country-level efforts to link research to action identifies rapid response units (units aimed at meeting policymakers' urgent needs for research evidence) as a strategy to aid `user-pull' efforts of knowledge translation [32]. `User-pull' efforts, it points out, are suited to situations in which the policymaker has identified a gap and seeks to address it in a timely manner.

We report here on the feasibility of one such rapid response mechanism in a low income country (Uganda), aimed at meeting policymakers' urgent needs for research evidence about health systems. At the time of this research, we were not aware of any other such mechanisms in a low income country. We were also aware of the fact that a survey of KT stakeholders in several low and middle income countries (LMICs) had suggested that such rapid response units would not be feasible or necessary in these settings [33].

### The rapid response service

Prior to this study, we developed a structure of a service to receive and respond to urgent requests for research evidence about health systems from policy and decision makers. We imagined that such a service would benefit mid to top level policy and decision makers at ministries of health, districts or local governments, Civil Society Organizations, health-related multi-, and bi-lateral agencies, the private sector, and legislators such as parliamentarians, among others. Such decision makers would not only have to be involved in making urgent policy decisions regularly, but they would also have to recognize and value research as an input in the policymaking process.

The service, although acknowledging the importance of all other elements of healthcare, especially clinical practice, defined its scope to include themes of health systems, for example, governance, delivery arrangements, and financial arrangements. The researchers had noted that although there still exist a number of challenges in actual completion, there has been a longer period of experience with evidence-based medicine as compared to its policy counterpart, driven especially by the nursing profession [34].

**Figure 1:**
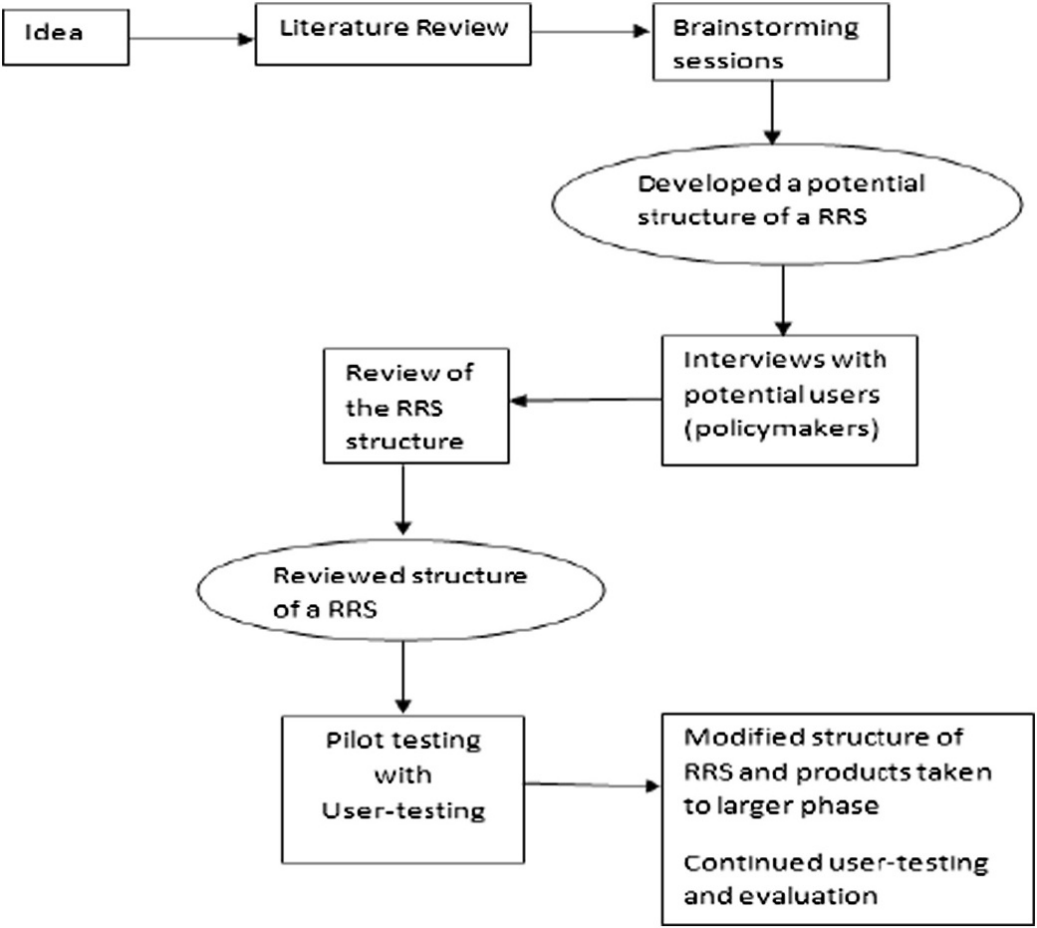
**Development of the REACH-PI rapid response service.**

Figure 1 summarizes the steps that we took to develop the structure. This involved a) reviewing the literature around such mechanisms as that proposed; b) brainstorming what the literature revealed; and c) then using this information to design a rapid response service. This design was presented to potential users through consultative interviews and their feedback used to modify it before piloting it.

The development process continued throughout the pilot period with activities that included provision of office space, recruitment of KT practitioners, the provision of easy internet access with subscription to relevant research databases, building networks with researchers, KT practitioners and policymakers (many of whom contributed to review processes), sensitization activities for policymakers, development of standard operating procedures outlining, among other things, the steps for producing a rapid response evidence brief, etc.

The service was structured as follows: The service was based at Makerere University, the oldest and largest university in Uganda located in Kampala, the capital of the country. The service was coordinated by hired staff that kept in regular contact with policymakers and health systems' stakeholders. They were supported by a wide network of researchers in and outside the region. The staff would receive questions from the policymakers by telephone, email or physical contact. They would then take the policymaker through a process of question clarification to ensure that the question was not only clear and asked in an answerable manner, but that it indeed fell within the scope handled by the service. Requests were rejected when they did not fall into the scope of the service in terms of topic or theme or urgency (where information was needed in more than 28 days). In search cases, the policymakers were politely directed to a source that the researchers felt would best handle the question. Following this, they searched for research evidence relevant to this query, appraised it, contextualized and summarized it. This summary would then be reviewed by local and international experts on the given subject. Such experts were identified through several processes - they could be authors from the literature reviewed, or experts identified by senior researchers on the study or through colleagues in other institutions like the WHO. Once the review process was complete, the staff prepared a short brief of usually four pages maximum, with clear key messages that would be submitted to the policymaker. (Where the available time was short, for example, with responses required in less than five days, an internal review was done by a senior researcher on the service. The brief would still be sent to an external reviewer and the policymaker informed that if the reviewer's input was substantially different from the brief delivered to him/her, an updated version would be provided as soon as possible). The above process would take any time less than 28 days. The service began with its scope limited to health systems questions concerning organizational arrangements, governance arrangements, strategies for implementing change, and financial arrangements. After the first six months, the scope was widened to include health technology assessments.

## Methods

### Design

Using a case study design, as guided by Yin [35], we studied the feasibility of such a mechanism as described above, to meet the urgent needs of policymakers for evidence about health systems. This was initially piloted with a few (12) policymakers for 8 months between March and October 2010, and was later run in a larger phase, open to all potential users, after January 2011. We report here on the findings between March 2010 and July 2012 (28 months).

### Population and setting

We offered the service to policymakers and stakeholders (including technical support staff, health managers, advocacy personnel, development partners) with varied backgrounds, endeavoring to include a wide range of potential users of such a service. These were from the Ministry of Health, Districts, Civil Society Organizations, health-related multi-, and bi-lateral agencies and the private sector. We involved mid and top level policy or decision makers, and their staffers.

During the first six months (pilot phase), we included a convenience sample of users policymakers who understood the principles of KT and this particular strategy of the rapid response service, starting with 3 participants and gradually increasing the number to 12. The service was later opened up to all policymakers and stakeholders in the health sector, with no sampling from the side of the researchers.

### Data collection

We collected data using questionnaires (questionnaires and other resources used on the service and for the study are attached as an Additional file 1 and can also be found in the REACH-PI [U] clearing house [36]); researchers on the team filled in responses to questions posed to the users on their particulars and those of the organizations with which they were affiliated. In addition, we collected data on how the service was used, and the immediate and delayed (after one month) experiences of the users following receipt of the rapid response evidence briefs. This was done every time there was a request for evidence. We also used key informant interviews guided by interview guides, for a purposive sample of policymakers (10) to better understand the process and their experience.

### Data analysis

We summarized the quantitative data using frequencies, proportions, and bar charts. In addition, we summarized the responses to the semi-structured questions, coding these and drawing common themes from them.

### Ethical issues

We received approval for this work from the School of Medicine Research and Ethics Committee of the College of Health Sciences, Makerere University. We also sought informed oral consent from each of the participants before they participated in the study. Participants' identities are kept confidential on the rapid response briefs. However, their information and particulars were left open to the research team to enable follow-up and in-depth contextual understanding of the process. Participants were informed about this.

## Results

The service was piloted between March and August 2010, and the open phase was taken on thereafter. Results included in this paper are from the first 28 months of the service, that is, March 2010 to June 2012. During this time, several staff worked on the service in both full time and part time capacity. Staff included researchers with a background in medical or social/population studies, coupled with research methods skills. In addition, they either had to have or get equipped with writing and policy analysis skills, and a general understanding of the health system and policy formulation process.

### Response to service

During the pilot phase, the service was offered purposively to 12 policy makers; of these 10 (83.3%) responded with the intention of using it if the opportunity arose. A total of 9 out of the 10 intending to use the service actually used it.

During the open phase, the service was open to all policymakers and stakeholders requiring health systems' evidence urgently. In total, 30 policymakers and stakeholders made use of the service. Table 1 shows the kinds of policymakers that used the service during the said time, while Table 2 shows the types of organizations with which they were affiliated. A total of 23 of the 30 users were from or attached to the Ministry of Health. Amongst these were those not working at the ministry but supporting the ministry, for example, as part of a technical working group. The majority, that is, 13 out of 23, identified themselves as mid-level policymakers at the Ministry of Health (MoH). Notably, no decision makers or stakeholders at the district level made use of the service during this time period.

**Table 1:** **Users of the rapid response service**

Type of policymakers	Number of policymakers
Senior policymaker in Ministry of Health	10
Mid-level policymaker in Ministry of Health	13
Decision maker in Non-Governmental Organization	5
Support staff to Ministry of Health	2
Total	30

**Table 2:** **Organization of affiliation of rapid response service users**

Organization of affiliation of policymakers	Number of policymakers
Ministry of Health	23
Bi/Multi-lateral Organizations	4
Government (Not Ministry of/health)	2
Non-Governmental Organizations	1
Districts	0
Total	30

These 30 policy makers and stakeholders generated 65 questions that were within the scope of the service (a list of the users and that of their questions can be found in the REACH-PI [U] clearing house [36]). One senior policymaker asked 7 questions, while a decision maker in a non-governmental organization asked 8 questions. The majority asked one or two questions each during this study period. There are also six instances in which a question was asked by more than one party, either separately or in a referral-style (one question asked by several persons in a sequential manner). One such instance included a question asked by a mid-level policymaker but following from the president of the republic of Uganda through a chain of referral of senior policymakers.

The topics or areas according to the scope are as shown in Table 3. The most prevalent (17/65) of the questions were about governance issues in the health system, followed by those about organizational arrangements (14 out of 65). Four questions in the group `other' included those on public health topics. Such questions did not fall in one of the categories that was identified as within the scope of the service at the time but were answered to allow the service test its capacity.

**Table 3:** **Topics or areas for research evidence needs posed to the rapid response service**

Type of question	Frequency
Governance	17
Organization	13
Health technology assessments	11
Implementation strategies	11
Financial arrangements	9
Other (E.g. Public health)	4
Total	65

The average duration in which answers were needed was 13 (12.8) days. While the modal time for response was 21 days, two questions required responses in 24 and 48 hours each and another six questions required response in 5 days. The maximum time that was given for a request was 28 days. Of the 65 questions, 81.5% (53 out of 65) responses were returned on time, 10.8% (7 out of 65) were returned later than the allotted time, and the 7.7% (5 out of 65) were not followed through to the end. Some of the reasons given for responses returned late were late reviews and deliveries through a third party, for example, a secretary. For those not followed through to the end, one of the reasons noted was that the policymakers were not available to clarify the question. This happened when the question was asked on behalf of another party, and the clarification was referred to the original policymaker. The fact that they were not available to ask the question themselves might mean that their availability to clarify it was also low.

**Figure 2:**
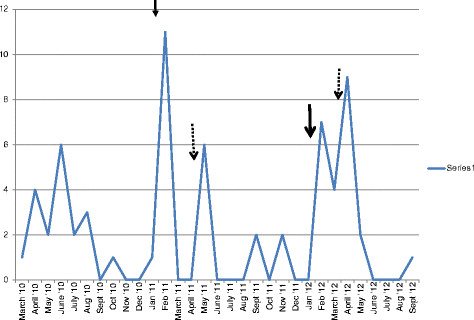
**Histogram showing the frequency of receipt of questions by the rapid response service.**

Figure 2 is a histogram showing the frequency with which the questions were received over the study period.

It is noted that there were periods in which there were sudden spurts in the number of questions. These periods represented times following active sensitization of policymakers about the service. There was active sensitization in February 2011 and January 2012, as marked by the solid arrows. Furthermore, there were more questions coming in the later part of the first half of the year. These times are marked out on the histogram with broken arrows.

A total of 11 of the questions were solicited for by the staff of the rapid response service. That is, the researchers prompted the policymakers to ask questions that they felt required evidence. All of the 11 were during the pilot phase; all questions after the pilot were unsolicited, that is, they were initiated by the policymakers.

The needs for which questions were asked varied. The commonest reason was to contribute to meetings and debates with peers, superiors, and development partners. Others included preparations for press conferences, input into strategic planning documents, advice to subordinates, proposals and concepts to management or development partners.

### Experience of users of the service

#### Before using the service

Through mechanisms provided for users to give feedback following receipt of a rapid response brief, users were able to give their experience of the briefs they received and the service in general. We compared this experience with that before the service. Before preparation of a brief, we asked the policymaker what they thought the answer to their question was and how confident they were about it. We also asked them what they would do without the rapid response brief and how confident they were in that course of action. Tables 4 and Table 5 show the confidence in answers and courses of action on a given decision before using the rapid response service. For 7 out of 65 (10.7%) questions, the policymaker was either confident or very confident of their answer before the brief and for 30 out of 65 (46.2%), they were neither confident nor `unconfident' in their own answers. For 3 out of 65 (4.6%) questions, policymakers had felt they had no idea what the answer to their question was before approaching the RRS.

**Table 4:** **Table showing confidence in respondents' own answers at the time of asking rapid response service help**

Confidence in answers before rapid response	Frequency	Percent
Very confident	1	1.5
Confident	6	9.2
Neither confident nor `unconfident'	30	46.2
`Unconfident'	19	29.2
Very `unconfident'	3	4.6
No idea	3	4.6
No response	3	4.6
Total	65	100.0

**Table 5:** **Table showing confidence in the respondents' course of action on given decision before rapid response service help**

Confidence in course of action before rapid response	Frequency	Percent
Very confident	1	1.5
Confident	11	16.9
Neither confident nor `unconfident'	26	40.0
`Unconfident'	18	27.7
Very `unconfident'	5	7.7
No idea	2	3.1
No response	2	3.1
Total	65	100.0

With or without an answer to their question at the time of requesting the rapid response service for help, policymakers would have to chart a course of action at some point. For only 12 out of 65 (18.4%) of questions were policymakers confident or very confident about their next steps of action. On two occasions, the policymakers reported they had no idea what their next steps would be without the rapid response service's help.

#### After using the service

Table 6 and Table 7 show the change in answers and courses of action following receipt of the rapid response briefs.

**Table 6:** **Table showing respondents' change in answers after using the rapid response brief**

Change in answer after rapid response brief	Frequency	Percent
Yes	43	66.2
No	14	21.5
No response	8	12.3
Total	65	100.0

**Table 7:** **Table showing change in respondents' course of action following rapid response brief**

Change in course of action following rapid response brief	Frequency	Percent
Yes	30	46.2
No	25	38.5
No response	10	15.4
Total	65	100.0

Following the briefs for 43 out of 65 (66.2%) of questions, the policymakers reported that their answer to the question had changed. For 28 out of 43 (65.1) questions for which answers changed, the policymakers reported feeling confident, while for 14 out of 43 (32.6%), they reported feeling very confident about their new answers.

For 30 out of 65 (46.2%) of questions posed to the service, the policymaker changed their course of action with the new information, while 25/65 (38.5%) continued with their previous course of action. For 15 out of 30 (50.0%) questions for which the course of action changed, the policymakers reported feeling confident, while for 14 out of 30 (46.7%), they reported feeling very confident about their new course of action.

### Satisfaction

Users of the service were also asked about satisfaction with the answers that they had received and with the service in general. In total, 42 (64.5%) were satisfied with the answers they received, while 16 out of 65 (24.6%) were very satisfied. In addition, for 29 out of 65 (44.6%) questions, the policymaker reported being satisfied with the service, while for 27 out of 65 (41.5%), they reported being very satisfied. For 2 out of 65 (3.1%), policymakers reported being unsatisfied with the service. On both occasions they reported that the rapid response brief had not been received within the agreed time.

For 7 out of 65 questions, there was loss-to-follow-up of the policymakers and therefore no feedback acquired. Reasons for loss-to-follow-up included policymakers leaving their post for another in a different location with no forwarding contact, or failing to reply to their known contacts. In addition, there was no feedback sought for those questions that were not followed through to the end.

### Attitudes

All policymakers involved agreed before and after use of the service that research evidence was an important part of the policymaking process. They however emphasized that it was only a part of several others and also cautioned that it was crucial that it not be `twisted' to fit one's needs.

### Challenges

A new start-up like the rapid response service is not without operational challenges. A major challenge we found was getting the policymakers to use the service at the start. Having had no experience with it, many potential users were skeptical. Another reason they reported for their skepticism was the fact that this was being run by researchers and they did not think that researchers understood their needs. Others showed no interest at all, possibly fuelled by the lack of awareness of the importance of evidence in decision making [25],[37]. We found that policymakers invariably required time, patience, and persistence in getting them interested in the service. However, once this happened, several began to refer their colleagues to it. It was also a challenge getting personnel with the right mix of skills and qualifications to work on the service, a challenge that has been previously reported in KT literature [9],[25],[30]. In addition, the demand on personal time that the service required was also a challenge for human resource. Other challenges that may especially apply to a low income country included fast and reliable internet connectivity, and access to databases and full text research papers [12].

## Discussion

Rapid response mechanisms to ease the barriers of timeliness and relevance of health systems and policy evidence for policymakers at the time they need it, are not a common phenomenon, especially in low income countries. What is usually seen when there is need for such evidence is specially convened task forces or think tanks of consultants and experts to address the given situation [38], or special committees and/or advisory bodies [39]. These are similar in a way to the rapid response service described in this paper, although they tend to work more in a reactive manner than proactively. Mechanisms that provide evidence in a relevant and timely manner are emerging in higher income settings and are increasingly popular for both clinical and policy needs [40]-[42]. For example, researchers on the Knowledge to Action (KTA) research program reported that their efforts to address knowledge users' needs for timely and user-friendly evidence in the Champlain region in Canada were highly valued by researchers and knowledge users [41]. Dobbins *et al*. and Hawkins *et al*. have also demonstrated that KT interventions that actively deliver and adapt to the needs of end users (ensuring the content of the message is relevant, timely and applicable to the intended user), rather than requiring them to access it independently, may lead to changes in knowledge and practice [43],[44].

Mechanisms working in a similar proactive manner have been considered for LMICs. A study to assess the level of interest in the establishment of a regional mechanism in Asia with the capacity to respond to questions from policy makers was done by Healy and colleagues [33]. They reported that such initiatives might not be feasible in these settings due to a number of reasons, including the fact that `the provision of information often depends on external information providers who cannot necessarily drop other commitments to give priority to a request'. They also cited the fact that readily available and specific information about health systems is in short supply and, when found, it has to be contextualized to a particular setting and system; and also the fact that evidence for health system policymaking requires careful and considered assessment, and planning within different contexts and within highly political environments. They noted that during such processes, clarifying what has been asked and shaping its answer is an iterative process, which would slow down the whole process. They further noted that such mechanisms may not be suited for health systems policy but for disaster planning and infectious disease management.

The findings of our study suggest that in fact the Healy paper findings more or less indicated a lack of experience with such initiatives and that when the challenges it cites are addressed, such a mechanism is feasible and accepted by policymakers. There are case studies of government support units or independent outside units that carry out KT activities in a manner similar to that described in this case study [8]. They are usually equipped with suitably skilled staff and appropriate infrastructure and have been known to assist in some of the barriers faced by policymakers in accessing and using research evidence for decisions and policy.

In this study, it was notable that the service was widely used by the ministry of health at the central headquarters and stakeholders in bilateral and multi-lateral agencies plus NGOs. There was notably no response from the districts. This may be a reflection of who feels they might benefit from such services, which would be mid-level to top administration policymakers in a health system who are generally involved in policymaking. In his paper, Nick Black [29] observes that the relation between research and policy depends on the arena and, therefore, the policymakers. He notes that research evidence is more influential in policymaking at the central level than the local level, the former being more characterized by negotiation and uncertainty than the latter. This would therefore be a reflection of different needs at different decision making levels and therefore the necessity to tailor the rapid response services differently, relevantly and suitably. However, the findings may also be because of the proximity of the ministry and where the service in this study was located, which promotes a closer relationship between the two. The studies by Innvaer S *et al*. [26] and Lavis *et al.*[28] noted that one facilitator of the uptake of research evidence during policymaking is close interaction between the two worlds. This interaction is also reflected in the fact that following a period of close interaction through sensitization activities, there was a spike in queries coming to the service.

There obviously was a need for information across the scope that was spelled out. There were, however, more governance and organization questions than finance questions. The fewer financial arrangement questions may reflect the fact that for the topic, policymakers may be looking to other areas for answers. For example, they may be consulting experts such as economists and international reports and publications, for example from the World Bank, and policymakers may rely on these more than the literature or such a rapid response service [31].

The findings in our study suggest that policymakers often knew what the answer to their question was but were not confident about it without the evidence. Furthermore, several had charted a course of action but were again not confident. With the evidence briefs, it was apparent that the confidence in their own answers improved, and so did the confidence in the courses of action. Campbell and colleagues note that policies and decisions based on evidence are more likely to give policymakers confidence in the decisions that they make [45]. In addition, although several had an idea as to what the answer to their query was, a change in answers and course of action reflects the fact that research evidence introduced more options for them to consider. This is in keeping with reflections from Strydom *et al*.'s paper, which asserts that scientific evidence indeed exposes policymaking to a wider range of validated concepts and experiences. This fact enables decisions and policies to be made based on a solid technical foundation and does open up a range of policy options for policymakers to consider and choose from [46].

In this study, policymakers echoed what several researchers have found, that policymakers are indeed interested in using research evidence and do value what it contributes to the policymaking process. Our findings also suggest that they were accepting of the service and generally found it satisfying to their needs. Judith Healy and colleagues' study found that policymakers and researchers were generally supportive of establishments similar to the rapid response service or some type of regional information mechanism, and that they in fact wished to be a part of its governance and other activities [33].

### Implications for future research

Our study did not attempt to profile several of the factors that may have an effect on the service, its operations and outcomes. Such factors may include the policy and decision makers that such a service can reach; its potential stakeholders, for example, funders; the staff working on it to tell what kind can make such a service work optimally; the environments or contexts in which the policymakers work and in which the services are placed. These are areas that future research may need to answer.

Future research may also consider work at the district and other local government levels to better understand what type of service is appropriate, considering that it is at the district level that decisions are implemented.

### Implications for policy

The findings from this research suggest a promising KT strategy. However, questions and concerns arise about the presence of capacity needed to run these services in low income countries. For example, the absence of reliable and fast internet connections or access to databases of research may hamper the optimal operations of such a service, and its sustainability. Authorities considering such a service would need to invest in the resources, including human resources, to be able to gain from it.

Furthermore, for many that are donor-funded like the service in this case study, there need to be plans for institutionalizing activities in order to ensure continuity even after donor funds are terminated. The service presented here will be absorbed into the Uganda National Health Research Organization at the end of the project period to ensure its sustainability. How and where it is institutionalized is important to ensure its continued impartiality and independence.

## Conclusion

Policymakers are sometimes faced with a barrier of timely and relevant research evidence when attempting to make evidence-informed policies and decisions. This barrier may further be amplified when decisions are to be taken in a short time, say hours or days. This research suggests that a rapid response mechanism is a feasible strategy to meet these urgent needs for research evidence for policy and decisions even in a low income country. Users of the service would be mainly those at the central level. This research suggests that the mechanism increases confidence of policymakers involved in the policy making process, and it provides them with more options for consideration during deliberation. While registering their satisfaction with using this service, policymakers emphasized the value of using research evidence for decision making. A rapid response mechanism for urgent evidence needs is feasible and acceptable to policymakers in low income countries.

## Authors' contributions

RM participated in the design and implementation of the study, and drafted the manuscript. ADO conceived of the study and participated in its design and implementation. UP participated in the design and implementation of the study. NKS participated in the conception, design and implementation of the study and participated in drafting the manuscript. All authors read drafts of the manuscript and approved the final manuscript.

## Additional file

## Electronic supplementary material

Additional file 1: Questionnaires and other resources used on the rapid response service.(PDF 518 KB)

Below are the links to the authors’ original submitted files for images.

Authors’ original file for figure 1

Authors’ original file for figure 2

Authors’ original file for figure 3

Authors’ original file for figure 4

Authors’ original file for figure 5

Authors’ original file for figure 6

Authors’ original file for figure 7

Authors’ original file for figure 8

Authors’ original file for figure 9
