# Supplementary material for: Feasibility of a rapid response mechanism to meet policymakers' urgent needs for research evidence about health systems in a low income country: a case study
Source: Implement Sci. 2014 Sep 10;9:114. doi: 10.1186/s13012-014-0114-z (PMC4172950; doi:10.1186/s13012-014-0114-z)
Supplement: Supplementary file 1 — Additional file 1: Questionnaires and other resources used on the rapid response service.(PDF 518 KB) [file 13012_2014_114_MOESM1_ESM.pdf]

Rapid response service resources

1. Operating procedures
2. Guides
   2.1. Question clarification guide
   2.2. Guide for searching, critically appraising and summarising evidence
   2.3. Guide for summarising and reporting responses to questions
3. Checklists and worksheets
   3.1. Assessment of a systematic review checklist
   3.2. Assessment of a primary study checklists
   3.3. Worksheets for summarising the findings
   3.4. Assessment of relevance of the research checklist
4. Forms
   4.1. User details form
   4.2. Initial contact form
   4.3. Question clarification form
   4.4. Search strategy form
   4.5. Internal review form (for assessing the response)
   4.6. External review form (for assessing the response)
5. Follow-up data collection forms
   5.1. Following preparation of response
   5.2. Following delivery of the response
   5.3. Second follow-up
6. Template for reporting responses to questions
7. Resources
   7.1. Databases
   7.2. Access to full-text articles and reports
   7.3. Sources of national data
8. Network of experts in Uganda
9. Network of experts outside of Uganda
10. Examples

1. Operating procedures for the pilot study

March to August 2010

Users

The pilot study will be offered to a limited number of participants; e.g.

- MoH
- District Health Officers
- NGOs

Selection criteria

1. Understands the purpose of the service and pilot
2. Likely to use the service at least once during the pilot study
3. Diverse (i.e. we will attempt to offer the service to people with diverse positions and backgrounds)

Selection process

- This will be a convenience sample selected informally
- We will start out with 3 people and gradually increase the number of users to 15 (or our capacity)

Description of participants

- We will collect information about each participant in the pilot for the first contact (only).

Staff

- 50% FTE (Project coordinator and other staff) will
  - contact users weekly and respond to calls and email messages
  - fill in initial contact form
  - assist with clarifying the question, searches and preparing reports
  - conduct follow-up interviews
- the team will also include the PI and Team leader who will also assist with clarifying the question, searches and preparing reports

Scope

The service will address questions about arrangements for organising, financing and governing health systems, and strategies for implementing changes.

Initial contact

- We will contact each user once weekly by email or telephone, as agreed with the user.
  - Set up schedule for calls / sending emails
- Each user will also be given an email address and telephone number that they can use to contact us.
- For each initial contact we will record the date, name of the person, whether the contact was initiated by the person or us, and the question (if any).
- If the contact was initiated by us or it is convenient, we will procede with question clarification. Otherwise, it will be decided who will clarify the question and when.

Question clarification

- Use question clarification from + guide.
- Should be done within 24 hours of initial contact.
- Reassess whether question is within the scope of the rapid response service.
- If it is agree on a timeline for responding.

Response preparation

- Decide who will prepare response, who will assist, who will sign off and timeline
- Use guide
- Complete search strategy form
- Conduct search
- Retrieve relevant material
- Assess relevant material (using checklists)
- Prepare summary of findings table
- Use template to prepare draft 3 page response
- Internal and external review of the 3 page response
  - Each report should be reviewed by at least 1 person internally (within the rapid response service team) and 1 external expert using review forms
  - External reviewers should be selected based on relevant expertise on the topic
  - Internal reviewers should focus primarily on the methods that were used and the extent to which the report appears to be appropriately prepared and sensible.
- Finalise the response
  - Respond to each comment by the reviewers
- Complete post-response preparation data collection form

Report delivery and post delivery data collection

- Deliver report to the person who asked the question electronically (or faxed or printed, if requested)
- Arrange follow-up interview
- Complete post-response data collection form (by email or phone, as agreed)
- Schedule second follow-up
  - To follow meeting at which the response will be discussed or a decision will be made
  - Find out when this will be
  - Generally the second follow-up should be within a couple of weeks

Data management

- A simple system will be set up for
    - Version numbers of forms, etc.
    - Filing of forms, etc. where they can be easily accessed (e.g. on an FTP server or on our personal computers) and ensuring that the correct versions are being used (e.g. if we keep them on our personal computers)
    - Filing completed forms
    - Transferring data to a spreadsheet or database

2. Guides

2.1 Question clarification guide

- The question should be clarified within 24 hours of the initial contact.

- Use question clarification form (3.3)

- **What type of question is it?**
  - Does the question concern **how big a problem is** (e.g. the prevalence of a risk factor or condition, limited access to a service, under or over-utilisation of a service)? If so, it is necessary to clarify the settings or populations of interest and the outcomes of interest (e.g. the risk factor, condition, access or utilisation)
  - Does the question concern **the causes of a problem** (e.g. why there is a high prevalence of a condition or why a service is not accessible)? If so, it is necessary to clarify the settings or populations of interest, exposures (suspected causes of the problem) and the outcomes of interest.
  - Does the question entail **identifying options to address a problem** (e.g. what the options are for improving delivery of an effective clinical intervention or what the options are for improving access to a service)? If so, it is necessary to clarify the settings or pouplations of interest, the range or types of interventions of interest (and any specific interventions of interest), and the outcomes of interest (including desired outcomes and any particular concerns about adverse effects or resource utilisation). What are the primary (most important) outcomes of interest and what are secondary outcomes?
  - Is the question about **the effects of a specific option** (or options) (e.g. how effective a specific programme or serviceis or what the advantages and disadvantages are of a change in who delivers a service or where it is delivered)? If so, it is necessary to clarify the settings or pouplations of interest, the specific interventions of interest (including what is currently being done), and the outcomes of interest (including desired outcomes and any specific concerns about adverse effects or resource utilisation). What are the primary (most important) outcomes of interest and what are secondary outcomes?
  - Is the question about **barriers to change** (e.g. reasons why an intervention is not being delivered or challenges to implementing a health system change)? If so, it is necessary to clarify the settings or pouplations of interest, any specific potential barriers or facilitators of interest, and the outcomes of interest (i.e. the desired change and any particular concerns about adverse effects or resource utilisation).
  - Does the question entail **identifying implementation strategies to address a problem** (e.g. what the options are for increasing utilisation of a service, improving adherence of health workers to guidelines, or changing a health system arrangement)? If so, it is necessary to clarify the settings or pouplations of interest, the range or types of interventions of interest (and any specific interventions of interest), and the outcomes of interest (including desired change and any particular concerns about adverse effects or resource utilisation). What are the primary (most important) outcomes of interest and what are secondary outcomes?
  - Is the question about **the effects of a specific implementation strategy** (or strategies) (e.g. how to increase utilisation of a service, how to improve health workers' adherence to a guideline, or how to change a health system arrangement)? If so, it is necessary to clarify the settings or pouplations of interest, the specific interventions of

interest (including what is currently being done), and the outcomes of interest (including desired changes and any specific concerns about adverse effects or resource utilisation). What are the primary (most important) outcomes of interest and what are secondary outcomes?
  - Is the quesion about **monitoring or evaluation** (e.g. how to monitor or evaluate the implementation of a health system change)? If so, it is necessary to clarify the settings or pouplations of interest, the specific intervention of interest (including what is being compared to; e.g. what is currently being done), and the outcomes of interest (including desired outcomes and any specific concerns about adverse effects or resource utilisation). What are the primary (most important) outcomes of interest and what are secondary outcomes?
  - If the question does not fit into any of the above categories and is within the scope of the rapid response service, how would you characterise the type of question?

- **What is the clarified question?**
  - The clarified question should specify the setting(s) and or population(s) of interest, the interventions or exposures if relevant, and the outcomes of interest in a single sentence.
  - This will form the basis for criteria to decide which research is relevant, developing a search strategy, and assessing the research that is found.
  - It is important to confirm with the user that the clarified quesiton is correct and that it has not been distorted by trying to focus it in this way.

- **Is the clarified question within the scope of the rapid response service?**
  - To be within the scope of the rapid response service the question should be about arrangehemts for organising, financing or governing a health system; or strategies for implementing changes.
    - Questions about clinical or public health interventions are generally outside of the scope of the service.
  - Questions that do not need to be answered within one month are also outside of the scope of the rapid response service.
  - If a question does not meet either of these two criteria, the reason should be noted and explained to the user.
  - If possible alternative sources of information or support should be suggested; e.g.
    - Sources of systematic reviews of the effects of clinical and public health interventions (The Cochrane Library and PubMed)
    - Sources of local or national data (based on the inventory that we will develop
  - Note any resources that would be helpful to the user and should be considered for inclusion in the clearing house that we will develop.

- **When is the response needed?**
  - Note the time and date by which a response is needed.
  - What is the reason for the deadline (e.g. a meeting, need to respond rapidly to something that was in the media or to a question from parliament, an emergency situation)
  - Agree on a time and date for delivering a response that is practical and meets the users needs.

- **What does the person asking the questions think the answer to the question is?**
  - After determining that a question is within the scope of the rapid response service and agreeing on a deadline, ask the user what he or she thinks the most likely answer is to the question without any more information.
  - Be as specific as possible and try to get as specific a response as possible, but do not push the user to guess.
    - If an answer is elicited, ask the user how confident she or he is regarding the answer using the following categories: Very confident, confident, neither confident or unconfident, unconfident, very unconfident.
  - What would be decided now without additional information?
    - If an answer is elicited, ask the user how confident she or he is regarding the answer using the following categories: Very confident, confident, neither confident or unconfident, unconfident, very unconfident.

2.2 Guide for searching, critically appraising and summarising evidence

Develop a search strategy

- Decide what type(s) of evidence are needed; e.g.
  - Local or national indicators (and comparators) to estimate how big a problem is (See Sources of national data (7.3) and the SURE guide for clarifying a problem.)
  - Qualitative research addressing how important a problem is to people or their perceptions of a problem (See SURE guide for clarifying a problem.)
  - Analyses of the causes of a problem (health systems research) (See the SURE guide for clarifying a problem.)
  - An analytic framework for identifying optionsn to address a problem (e.g. in a systematic review or an overview of reviews) (See the SURE guide for deciding on and describing options.)
  - Systematic reviews of the effects of options (See the SURE guide for deciding on and describing options.)
  - Qualiative or quantitative studies of barriers to change and facilitators (See SURE guide for identifying and addressing barriers to implementing the options.)
  - An analytic framework for identifying implementation strategies (See SURE guide for identifying and addressing barriers to implementing the options.)
  - Systematic reviews of the effects of implementation strategies
  - Research or methodology related to monitoring and evaluation (See SURE guide for clarifying uncertainties, and needs and priorities for monitoring and evaluation.)
- Decide what databases or other sources to search; e.g.
  - For local or national indicators (and comparators) - Sources of national data (7.3)
  - For qualitative research – PubMed and experts in Uganda
  - For analyses of the causes of a problem – PubMed and experts
  - For analytic frameworks for identifying optionsn to address a problem – experts, a systematic review or overview of reviews, and PubMed
  - For systematic reviews of the effects of options – PPD/CCNC database, The Cochrane Library or PubMed
  - For studies of barriers to change and facilitators - PubMed
  - For analytic framework for identifying implementation strategies – experts, a systematic review or overview of reviews, and PubMed
  - Systematic reviews of the effects of implementation strategies – cadth Rx for Change, The Cochrane Library, and PubMed
  - For research or methodology related to monitoring and evaluation – PubMed, Cochrane Methods Register, and methodologists
- Develop a search strategy using boolean logic including text words and keywords for
  - (settings or populations of interest – connected with OR) AND
  - (interventions of interest, if relevant – connected with OR) AND
  - (the outcomes of interest – connected with OR) AND
  - (the types of research of interest – e.g. using hedges in PubMed
- Use relevant articles to identify search terms, to search for ‘Related articles’ in PubMed, or to search for articles that cite key references (e.g. using Google Citation).
- Narrow or broaden the search strategy if there are too many or too few hits
- Screen the hits, flagging articles likely provide relevant evidence and should be retrieved, that likely provide useful background information (e.g. an analytic framework) and should be retrieved, that possibly provide relevant evidence or background and should only be retrieved if there is need and sufficient time, and not relevant articles.

- Search for systematic reviews and overviews of systematic reviews first. Only search for primary studies if a good quality, up-to-date review cannot be found.
- Critically appraise the reliability of the evidence using a checklist for assessing a systematic review (3.1) or a primary study (3.2)
- Prepare a summary of findings for the most relevant and reliable evidence using the Worksheets for summarising the findings (3.3)

2.3 Guide for summarising and reporting responses to questions

- Use the SURE rapid response template
- The title should reflect the clarified **question** that was asked.
- The **Background** should not be more than one or two paragraphs
  - It should clarify the motivation for asking the question / preparing the rapid response.
  - It should only provide key important background information that is essential for the response to be understandable, including explanation of whichever of the following is not obvious or may be confusing, if not explained:
    - The people, settings or problem
    - The intervention(s) or policies
    - The comparison
    - The outcomes or goals of the interventions or policies
  - The background should not repeat information in the summary of findings
- The **key messages** should be succinct and summarise
  - There should not be more than 4 or 5 key messages summarising the most important messages from the summary of findings + a key message regarding the relevance of the review.
  - The key messages should not extend beyond page 1.
- The **summary of findings**
  - If necessary, subheadings can be used; e.g. if there are several key comparisons or if the question can best be answered by splitting it up into two or more subquestions.
  - The first paragraph should be one or two sentences summarising the key information from the 'about the review' table on the last page; e.g. the total number of included/relevant studies and where they were done or the specific types of interventions for which studies were found.
  - The second paragraph should provide any information that is necessary to understand the findings. It should not repeat what is in the background and should not include details about study designs.
  - The bullet points should highlight the key findings from the summary of findings table.
  - These should be qualitative statements using wording that is consistent with what is suggested at the end of the Worksheets for summarising the findings (3.3), adapted from guidance for Cochrane plain language summaries.
  - Do not say "no difference"!
- The **summary of findings table** should be prepared using the Worksheets for summarising the findings.
  - If the table is largely empty (e.g. no studies) or not informative (e.g. only very low quality evidence), it should be deleted.
  - Ideally, it should use the standard format. However, the format can be changed, if that would clarify the findings; e.g. a column for comments can be added or the 'Impacts' column can be relabled (and if needed split) if the findings can be reported in a standard way across outcomes.
- **Relevance of the research to the question being asked**
  - The findings column should state in as few words as possible the evidence (or lack of evidence) that provides the basis for the interpretation. (See SUPPORT Summaries for examples.)
  - The interpretations should be guided by the Assessment of relevance of the research checklist.

- **About the research underlying this response**
  - Delete or relable rows that are not relevant.
  - The ”What we searched for” column should clarify the selection criteria that were used. If the Response is based on a single systematic review, the heading can be changed to ”What the review authors searched for” and the column should reflect the selection criteria for the review.
  - The ”What we found column should summarise the characteristics of the studies that were found. If relevant include the number of studies for each different type of
    - intervention
    - participants
    - setting (e.g. country)
    - primary outcome reported
    - study design
  - The date of most recent search should either be when searches were conducted for the response or, if the response is based on a single systematic review, when the searches for the review were conducted.
  - Limitations should be based on the ”Assessment of a systematic review” checklist (3.1) and state succinctly either the limiations of the review that was done for the response or, if the response is based on a single systematic review, limitations of the systematic review.
    - This should either say “This Response is based on a systematic review with only minor limitations.” OR
    - Any important limitations should be noted. For example,
      - “This is a reliable systematic review with only minor limitations. However, it has not been updated since 1999.” OR
      - “This was an exhaustive review of the available research, but few rigorous evaluations were found.” OR
      - ”We were unable to find a systematic review that addresses this question. Therefore this response is not based on a systematic review and it was not possible to conduct an exhaustive search for relevant research.
- **References**
  - This should include the systematic review(s) and any primary studies (not included in a systematic review) that met the selection criteria and were assessed.
  - If relevant add a subheading, such as ”**Related literature**”, and include key references for information that is helpful to understand the problem, provides details about the interventions, or helps to put the results of the Response in a broader context.
- **Conflicts of interest**
  - Typically, this should say ”None declared.” or ”None known.” or ”None.”
- **Acknowledgements**
  - Include the people who peer reviewed the Response and anyone that was consulted in preparing the response, provided they gave permission to be acknowledged.
- **For more informatin contact**
  - This can be the name and email address of the person who prepared the Response, a fixed email address for the Rapid Response Service, or the person responsible for the Rapid Response Service.

3. Checklists and worksheets

3.1 Assessment of a systematic review checklist

- Initially we can use the SURE checklist.
- We may want to simplify it and adapt it for flagging important limitations when we are unable to find a reliable, up-to-date systematic review and do our own review of primary research.

3.2 Assessment of a primary study checklists

- Initially we can use the EPOC criteria.
- We may want to simplify these, use a generic checklist that can be applied to different study designs, or add checklists for other relevant study designs.

EPOC risk of bias criteria for studies with a separate control group (randomised trials, non-randomised trials, controlled before-after studies)

Nine standard criteria are used. Further information can be obtained from the Cochrane handbook section on Risk of Bias and from the draft methods paper on risk of bias under the EPOC specific resources section of the EPOC website.

Was the allocation sequence adequately generated?
Score "Yes" if a random component in the sequence generation process is described (eg Referring to a random number table). Score "No" when a nonrandom method is used (eg performed by date of admission). CCTs and CBAs should be scored "No". Score "unclear" if not specified in the paper.

Was the allocation adequately concealed?
Score "Yes" if the unit of allocation was by institution, team or professional and allocation was performed on all units at the start of the study; or if the unit of allocation was by patient or episode of care and there was some form of centralised randomisation scheme, an on-site computer system or sealed opaque envelopes were used. CBAs should be scored "No". Score "unclear" if not specified in the paper.

Were baseline outcome measurements similar?*
Score "Yes" if performance or patient outcomes were measured prior to the intervention, and no important differences were present across study groups. In RCTs, score "Yes" if imbalanced but appropriate adjusted analysis was performed (e.g. Analysis of covariance). Score "No" if important differences were present and not adjusted for in analysis.** If RCTs have no baseline measure of outcome, score "Unclear".**

Were baseline characteristics similar?
Score "Yes" if baseline characteristics of the study and control providers are reported and similar. Score "Unclear" if it is not clear in the paper (e.g. characteristics are mentioned in text but no data were presented). Score "No" if there is no report of characteristics in text or tables or if there are differences between control and intervention providers. Note that in some cases imbalance in patient characteristics may be due to recruitment bias whereby the provider was responsible for recruiting patients into the trial.

Were incomplete outcome data adequately addressed?*

Score "Yes" if missing outcome measures were unlikely to bias the results (e.g. the proportion of missing data was similar in the intervention and control groups or the proportion of missing data was less than the effect size i.e. unlikely to overturn the study result). Score "No" if missing outcome data was likely to bias the results. Score "Unclear" if not specified in the paper (Do not assume 100% follow up unless stated explicitly).

Was knowledge of the allocated interventions adequately prevented during the study? *

Score "Yes" if the authors state explicitly that the primary outcome variables were assessed blindly, or the outcomes are objective, e.g. length of hospital stay. Primary outcomes are those variables that correspond to the primary hypothesis or question as defined by the authors. Score "No" if the outcomes were not assessed blindly. Score "unclear" if not specified in the paper.

Was the study adequately protected against contamination?

Score "Yes" if allocation was by community, institution or practice and it is unlikely that the control group received the intervention. Score "No" if it is likely that the control group received the intervention (e.g. if patients rather than professionals were randomised). Score "unclear" if professionals were allocated within a clinic or practice and it is possible that communication between intervention and control professionals could have occurred (e.g. physicians within practices were allocated to intervention or control)

Was the study free from selective outcome reporting?
Score "Yes" if there is no evidence that outcomes were selectively reported (e.g. all relevant outcomes in the methods section are reported in the results section). Score "No" if some important outcomes are subsequently omitted from the results. Score "unclear" if not specified in the paper.

Was the study free from other risks of bias?
Score "Yes" if there is no evidence of other risk of biases

* If some primary outcomes were imbalanced at baseline, assessed blindly or affected by missing data and others were not, each primary outcome can be scored separately.

**If "UNCLEAR" or "No", but there is sufficient data in the paper to do an adjusted analysis (e.g. Baseline adjustment analysis or Intention to treat analysis) the criteria should be re scored to "Yes".

Risk of bias for interrupted time series studies

Seven standard criteria are used. Further information can be obtained from the Cochrane handbook section on Risk of Bias and from the draft methods paper on risk of bias under the EPOC specific resources section of the EPOC website.

Note: If the ITS study has ignored secular (trend) changes and performed a simple t-test of the pre versus post intervention periods without further justification, the study should not be included in the review unless reanalysis is possible.

Was the intervention independent of other changes?
Score "Yes" if there are compelling arguments that the intervention occurred independently of other changes over time and the outcome was not influenced by other confounding variables/historic events during study period. *If Events/variables identified, note what they are.* Score "NO" if reported that intervention was not independent of other changes in time.

Was the shape of the intervention effect pre-specified?
Score "Yes" if point of analysis is the point of intervention OR a rational explanation for the shape of intervention effect was given by the author(s). Where appropriate, this should include an explanation if the point of analysis is NOT the point of intervention;Score "No" if it is clear that the condition above is not met

Was the intervention unlikely to affect data collection?

Score "Yes" if reported that intervention itself was unlikely to affect data collection (for example, sources and methods of data collection were the same before and after the intervention); Score "No" if the intervention itself was likely to affect data collection (for example, any change in source or method of data collection reported).

Was knowledge of the allocated interventions adequately prevented during the study?***

Score "Yes" if the authors state explicitly that the primary outcome variables were assessed blindly, or the outcomes are objective, e.g. length of hospital stay. Primary outcomes are those variables that correspond to the primary hypothesis or question as defined by the authors. Score "No" if the outcomes were not assessed blindly. Score "unclear" if not specified in the paper.

Were incomplete outcome data adequately addressed?***

Score "Yes" if missing outcome measures were unlikely to bias the results (e.g. the proportion of missing data was similar in the pre- and post-intervention periods or the proportion of missing data was less than the effect size i.e. unlikely to overturn the study result). Score "No" if missing outcome data was likely to bias the results. Score "Unclear" if not specified in the paper (Do not assume 100% follow up unless stated explicitly).

Was the study free from selective outcome reporting?

Score "Yes" if there is no evidence that outcomes were selectively reported (e.g. all relevant outcomes in the methods section are reported in the results section). Score "No" if some important outcomes are subsequently omitted from the results. Score "unclear" if not specified in the paper.

Was the study free from other risks of bias?

Score "Yes" if there is no evidence of other risk of biases.
e.g. should consider if seasonality is an issue (i.e. if January to June comprises the pre-intervention period and july to December the post, could the "seasons' have caused a spurious effect).

*** If some primary outcomes were assessed blindly or affected by missing data and others were not, each primary outcome can be scored separately.

3.3 Worksheets for summarising the findings

- Initially we can use the SURE checklist.
- We may want to simplify it and adapt it for flagging important limitations when we are unable to find a reliable, up-to-date systematic review and do our own review of primary research.

3.4 Assessment of relevance of the research checklist

Adapted from SUPPORT Tools for Evidence-Informed Policymaking (STP) and the SURE guides

Applicability

1. Was the research conducted in the setting of interest or were the findings consistent across diverse settings or time periods?

If not, are there important differences between the setting(s) where the research was done and the setting(s) of interest in:

2. Health system arrangements, such that the intervention could not work in the same way?
3. On-the-ground realities and constraints that might substantially alter the feasibility, acceptability or potential impacts of the intervention?
4. Are there important differences in the baseline conditions that might yield different absolute effects even if the relative effectiveness was the same?

Equity

1. Are there plausible reasons for anticipating differences in the relative effectiveness of the option for disadvantaged groups or settings?
2. Are there likely to be different baseline conditions across groups or settings such that that the absolute effectiveness of the option would be different, and the problem more or less important, for disadvantaged groups or settings?
3. Are there important considerations that should be made when implementing the option in order to ensure that inequities are reduced, if possible, and that they are not increased?

Costs (resource use)

1. What are the most important costs, including the costs of implementing and sustaining the option?
2. What information is there about those costs, either from a systematic review or other sources?
3. Is there important uncertainty about medium to long term costs?
4. Is there important uncertainty about the applicability of any reported costs?

Monitoring and evaluation

1. Is monitoring necessary?
2. If monitoring is necessary, what should be measured?
3. Is an impact evaluation necessary?
4. If an impact evaluation is necessary, what should be evaluated and how?

4. Forms

4.1 User details form

The following information should only be collected once for each user:

Name:

Email address:

Telephone:

Position (specify + check one of the following):

- ☐ senior policymaker in MoH
- ☐ mid-level policymaker in MoH
- ☐ support staff in MoH
- ☐ District Health Officer
- ☐ decision maker in NGO
- ☐ support staff in NGO
- ☐ other (specify)

Organisation:

Background (check all that apply):

- ☐ Medical doctor
- ☐ Other health profession (specify)
- ☐ Research training (MSc or PhD)
- ☐ Research experience (specify number of years)

4.2 Initial contact form

Date:

Name of person who is filling in this form:

Name of person contacted or asking the question:

Get user details (Data collection form 4.1), if not previously collected.

Who initiated the contact?

- ☐ We did
- ☐ S/he did

What is the question being asked?

- ☐ None
- ☐ Note the question in the words of the person asking it:

Is the question within the scope of the rapid response service?

- Is the question about arrangements for organising, financing and governing health systems, or strategies for implementing changes?
  ☐ Yes
  ☐ No (note any advice given to the person asking the question and any comments about the service and being told that the question is not within our scope and stop here)

- Is an answer needed within hours or days?
  ☐ Yes
  ☐ No (note any advice given to the person asking the question and any comments about the question not needing a rapid response and stop here)

If the question is within the scope of the rapid response service, who will clarify the question and when?

4.3 Question clarification form

See Question clarification guide (2.1)

Date:

Name of person who is helping to clarify the question:

Name of person asking the question:

Get user details (Data collection form 3fi), if not previously collected.

If the person is asking the question on behalf of someone else or in response to someone else, who initially asked the question?

Name of person asking the question:

Position (specify + check one of the following):

- ☐ senior policymaker in MoH
- ☐ mid-level policymaker in MoH
- ☐ support staff in MoH
- ☐ District Health Officer
- ☐ decision maker in NGO
- ☐ support staff in NGO
- ☐ other (specify)

1. What is the initial question that was asked?

2. Why is the question being asked?

3. What decision or action will the answer inform?

4. When is an answer is needed?

5. Why is the answer needed within hours or days?

6. What type of question is it? Check all that apply:
   - ☐ Assessment of the size of a problem
   - ☐ Assessment of the causes of a problem
   - ☐ Identification of options to address a problem
   - ☐ Assessment of one or more options
   - ☐ Assessment of the need for monitoring or evaluation
   - ☐ Identification of barriers to implementation
   - ☐ Identification of implementation strategies
   - ☐ Assessment of one or more implementation strategies
   - ☐ Assessment of the need for monitoring or evaluation
   - ☐ Other (specify)

7. What is the population or setting of interest?

8. What types of interventions or exposures (if any) are of interest?

9. What are the outcomes of interest?

10. What is the clarified question?

11. Is the clarified question within the scope of the rapid resonse service?

   - Is the question about arrangements for organising, financing and governing health systems, or strategies for implementing changes?
     ☐ Yes
     ☐ No (note any advice given to the person asking the question and any comments about the service and being told that the question is not within our scope and stop here)

   - Is an answer needed within hours or days?
     ☐ Yes
     ☐ No (note any advice given to the person asking the question and any comments about the question not needing a rapid response and stop here)

   - If a question does not meet either of these two criteria, the reason should be noted and explained to the user.

   - If possible alternative sources of information or support should be suggested and any resources that should be included in the clearing house that we will develop should be noted.

12. When is the response needed?

   Time and date by which a response is needed:

   Reason for the deadline:

   Agreed upon time and date for delivering a response:

13. What does the person asking the questions think the answer to the question is?

14. How confident is s/he regarding the assumed answer?
   ☐ Very confident
   ☐ Confident
   ☐ Neither confident or unconfident
   ☐ Unconfident
   ☐ Very unconfident.

15. What would be decided now without additional information?

16. How confident is s/he regarding the decision?
- ☐ Very confident
- ☐ Confident
- ☐ Neither confident or unconfident
- ☐ Unconfident
- ☐ Very unconfident.

4.4 Search strategy form

Date:

Name of the person doing the search:

Question:

What type(s) of evidence are needed?
☐ local or national indicators
☐ comparators
☐ systematic review(s)
☐ overview of reviews
☐ primary quantitative research (specify relevant designs)
☐ qualitative research
☐ indicators
☐ other (specify)

What databases or other sources should be searched (and, if relevant, in what order)?
☐ Sources of national data (7.3)
☐ PPD/CCNC database
☐ The Cochrane Library
☐ PubMed
☐ cadth Rx for Change
☐ Google scholar (citation searching)
☐ PubMed related articles
☐ Other databases (specify)
☐ Experts in Uganda (specify)
☐ Experts outside of Uganda (specify)
☐ Other sources (specify)

What terms should be used when searching databases?
☐ settings or populations of interest (list relevant terms)

☐ interventions of interest (list relevant terms)

☐ outcomes of interest (list relevant terms)

☐ types of research of interest (specify hedges in PubMed or list relevant terms)

Prepare a record of all of the search strategies that were used, including search terms.

For each source that is searched note the number of hits that are screened and number that are included.

4.5 Internal review form (for assessing the response)

Title (question):

Name of internal reviewer:

Date:

☐ Is the question that the Response addresses clear and sensible?

☐ Is the background succinct and informative?

> ☐ Is the motivation for the question and/or Response clear?
>
> ☐ Is there superfluous information that should be removed or unclear information that should be edited?
>
> ☐ Is any background information that is needed to make sense of the key messages and the findings missing?

☐ Are the key messages supported by the research that was reviewed, do they address the question and are they informative?

> ☐ Are there key messages that should be removed or edited?
>
> ☐ Are there key messages that are missing?

☐ Is the text describing the findings succinct and informative?

> ☐ Is there superfluous information that should be removed or unclear information that should be edited?
>
> ☐ Is any important information missing that is needed to understand the findings?

☐ Are the key findings well summarised as bullet points?

> ☐ Are they expressed using words that are consistent with the recommended terms in the worksheets for summarising the evidence?
>
> ☐ Are there three or fewer bullet points? If not should any of the bullet points be removed?
>
> ☐ Are there critical findings that are missing and should be added?

☐ Are the main results summarised in a summary of findings table?

> ☐ If so, is the table understandable and consistent with the recommended format? If not, should it be edited?

☐ Are unimportant outcomes included that should be removed?

☐ Are any critical outcomes missing that should be added?

Are the judgements that are made about the relevance of the research sound and do they flow from the findings? If not, should they be edited?

☐ Is there superfluous information that should be removed?

☐ Are there important considerations that are missing and should be added?

☐ Is the description of the underlying research (what was searched for and what was found) appropriate?

☐ Is there unclear information that should be edited or superfluous information that should be removed?

☐ Is there important information that is missing?

☐ Are the references complete?

☐ Are the names of the people who prepared the Response reported?

☐ Are any conflicts of interest reported?

☐ Are the people who were consulted or reviewed a draft of the response acknowledged?

☐ Is the citation complete and correct?

☐ Is an email address provided for further information?

Is the 'audit trail' for the Response complete and adequate, including:
☐ User details form
☐ Initial contact form
☐ Question clarification form
☐ Search strategy form
☐ Assessment of a systematic review checklist (if relevant)
☐ Assessment of a primary study checklists (if relevant)
☐ Worksheets for summarising the findings
☐ Assessment of relevance of the research checklist
☐ External review form
☐ Internal review form (this form)

4.6 External review form (for assessing the response)

Dear Dr. [*Name*],

We would be grateful if you could review the attached SURE Rapid Response within the next X days. The Response addresses the following question:

XXX?

If you are unable to review the attached Response, please let me know as soon as possible. We would appreciate it if you could suggest other people who are knowledgeable about the topic of this Response, if you are not able to review it yourself.

SURE Rapid Responses are prepared to address policymakers and managers needs for research evidence when appraised and contextualised evidence is needed in a matter of hours or days, if it is going to be of value to them. The Responses address questions about arrangements for organising, financing and governing health systems, and strategies for implementing changes. We currently are conducting a pilot study of the SURE Rapid Response Service.

We would appreciate your advice regarding the following and any specific suggestions you have for improving the Response, particularly the **key messages** and the section of the summary that addresses the **relevance** of the research:

General

1. Are you aware of any research that addresses the same question that is not included in the Response and should have been?

Key messages

2. Are the key messages clear, informative and consistent with the research findings?
3. Are there any changes that you would suggest to the background or key messages?

Summary of findings

4. Is the summary of the research findings clear, relevant and appropriate?

Relevance

5. Are the interpretations that are made appropriate, relevant and likely to be helpful to policymakers and managers?
6. Are there any additional comments or specific changes that you would suggest regarding applicability, equity, costs, or monitoring and evaluation?

Description of the underlying research

7. Does the box describing the underlying research ('About the research underlying this summary') clearly describe what was searched for and found?

Additional information

8. Is there any other literature that addresses this question that you think would be particularly useful to policymakers and managers, including related systematic reviews, information that is helpful to understand the problem, provides details about the interventions, or helps to put the results of the research in a broader context?

9. Is it OK to acknowledge you for reviewing this summary?

Thank you for considering,

5. Follow-up data collection forms

5.1 Following preparation of response

Date:

Name of person(s) filling out the form:

Name of person(s) who clarified the question and prepared the response:

Record any observations or comments you have regarding the following:

1. Was the question clearly within the scope of the service?
   ☐ Yes
   ☐ No
   ☐ Not sure

2. Did the question clearly need to be answered within hours or days?
   ☐ Yes
   ☐ No
   ☐ Not sure

3. Was this an appropriate use of the service?
   ☐ Yes
   ☐ No
   ☐ Not sure

4. How confident are you that the answer provided was an appropriate answer to the question that was asked (and why)?
   ☐ Very confident
   ☐ Confident
   ☐ Neither confident or unconfident
   ☐ Unconfident
   ☐ Very unconfident.
   Why were you confident or unconfident that the answer was appropriate?

5. How satisfied are <u>you</u> with the <u>answer</u> that was provided to the question?
   ☐ Very satisfied
   ☐ Satisfied
   ☐ Neither satisfied or unsasisfied (specify why)
   ☐ Unsatisfied (specify why)
   ☐ Very unsatisified (specify why)

6. How satisfied are <u>you</u> with how the <u>service</u> responded to the person asking the question?
   ☐ Very satisfied
   ☐ Satisfied
   ☐ Neither satisfied or unsasisfied (specify why)
   ☐ Unsatisfied (specify why)
   ☐ Very unsatisified (specify why)

7. What were the strengths of the service in terms of how this particular question was answered?

8. What were the weaknesses of the service in terms of how this particular question was answered?

9. How could the service be improved from the perspective of the user?

10. How could the service be improved from the perspective of the people responding (including improvements to the procedures, resources, support, etc.)?

5.2 Following delivery of the response

Date:

Name of interviewer:

Name of person being interviewed:

1. What does the person who asked the questions think the answer to the question is now? (Has this changed from before receiving the response?)

2. How confident is s/he regarding the <u>answer</u>?
   ☐ Very confident
   ☐ Confident
   ☐ Neither confident or unconfident
   ☐ Unconfident
   ☐ Very unconfident.

3. What would be decided now with the additional information?

4. How confident is s/he regarding the <u>decision</u>?
   ☐ Very confident
   ☐ Confident
   ☐ Neither confident or unconfident
   ☐ Unconfident
   ☐ Very unconfident

5. How satisfied is s/he with the <u>answer</u>?
   ☐ Very satisfied
   ☐ Satisfied
   ☐ Neither satisfied or unsasisfied (specify why)
   ☐ Unsatisfied (specify why)
   ☐ Very unsatisified (specify why)

6. How satisfied is s/he with the <u>service</u>?
   ☐ Very satisfied
   ☐ Satisfied
   ☐ Neither satisfied or unsasisfied (specify why)
   ☐ Unsatisfied (specify why)
   ☐ Very unsatisified (specify why)

7. Does the s/he have any suggestions for how the service could be improved?

8. How important would s/he say it is that health system decisions are informed by research evidence?

☐ Very important
☐ Important
☐ Neither important or unimportant (specify why)
☐ Unimportant (specify why)
☐ Very unimportant (specify why)

9. What would s/he say the role of research evidence is in informing health system decisions?

10. How important would s/he say it is that REACH/EVIPNet is?
☐ Very important
☐ Important
☐ Neither important or unimportant (specify why)
☐ Unimportant (specify why)
☐ Very unimportant (specify why)

11. What would s/he say the role of REACH/EVIPNet is?

5.3 Second follow-up

Date:

Name of interviewer:

Name of person being interviewed:

1. What does the person who asked the questions think the answer to the question is now? (Has this changed since first getting the response?)

2. How confident is s/he regarding the <u>answer</u> now?
   ☐ Very confident
   ☐ Confident
   ☐ Neither confident or unconfident
   ☐ Unconfident
   ☐ Very unconfident.
   Has this changed?

3. What decision was made?

4. How confident is s/he regarding the <u>decision</u> now?
   ☐ Very confident
   ☐ Confident
   ☐ Neither confident or unconfident
   ☐ Unconfident
   ☐ Very unconfident
   Has this changed?

5. What, if any, additional information was obtained that was important and may have affected how confident s/he is with the answer or the decision?

6. How satisfied is s/he with the <u>answer</u> now?
   ☐ Very satisfied
   ☐ Satisfied
   ☐ Neither satisfied or unsasisfied (specify why)
   ☐ Unsatisfied (specify why)
   ☐ Very unsatisified (specify why)
   Has this changed?

7. How satisfied is s/he with the <u>service</u> now?
   ☐ Very satisfied
   ☐ Satisfied
   ☐ Neither satisfied or unsasisfied (specify why)
   ☐ Unsatisfied (specify why)
   ☐ Very unsatisified (specify why)
   Has this changed?

8. Does the s/he have any suggestions for how the service could be improved?

9. How important would s/he say it is that health system decisions are informed by research evidence?
   ☐ Very important
   ☐ Important
   ☐ Neither important or unimportant (specify why)
   ☐ Unimportant (specify why)
   ☐ Very unimportant (specify why)
   Has this changed?

10. What would s/he say the role of research evidence is in informing health system decisions? (Has this changed?)

11. How important would s/he say it is that REACH/EVIPNet is?
    ☐ Very important
    ☐ Important
    ☐ Neither important or unimportant (specify why)
    ☐ Unimportant (specify why)
    ☐ Very unimportant (specify why)
    Has this changed?

12. What would s/he say the role of REACH/EVIPNet is? (Has this changed?)

6. Template for reporting responses to questions

- This has been adapted from the template for SUPPORT Summaries and is in a separate .dot file.

7. Resources (including databases, full-text articles and reports, and sources of national data)

7.1 Databases (from STP 7)

**PPD/CCNC - database www.researchtopolicy.ca/search/reviews.aspx**
Features<br>• Accessible online at no cost<br>• Focused exclusively on governance, financial and delivery arrangements within health systems<br>• Contains Cochrane reviews of impacts, other reviews of impacts, and reviews that address other types of questions (e.g. reviews of qualitative studies), as well as overviews of systematic reviews and policy briefs<br>• Provides links to user-friendly summaries (when they exist) and to scientific abstracts<br>***What is in it?***<br>• Systematic reviews that address any type of question about governance, financial and delivery arrangements within health systems<br>• Overviews that identify and synthesise the many systematic reviews that address a specific health systems issue or challenge<br>***How can it be searched?***<br>• Type of governance, financial and delivery arrangement (by clicking on the relevant category)<br>• Type of systematic review, namely review of impacts, Cochrane review of impacts, and review addressing another type of question<br>• Type of overview, namely policy brief written primarily for policymakers and overview of systematic reviews written primarily for researchers<br>***What resources are provided for search results?***<br>• Link(s) to a user-friendly summary that highlights decision-relevant information (if available)<br>– **Australasian Cochrane Centre (ACC) Policy Liaison Initiative** (primarily for policymakers in Australia)<br>– **Database of Abstracts of Reviews of Effects (DARE)** (primarily for healthcare providers but no limitations per se)<br>– **Effective Health Care Research Programme Consortium** (primarily for healthcare providers and policymakers in low- and middle-income countries)<br>– **Health-evidence.ca** (primarily for public health practitioners and policymakers)<br>– **Reproductive Health Library** (primarily for reproductive health practitioners and policymakers)<br>– **Rx for Change** (primarily for policymakers interested in influencing prescribing behaviour or healthcare provider behaviour more generally)<br>– **SUPPORT** (primarily for policymakers in low- and middle-income countries)<br>• Link(s) to a scientific abstract (when available)<br>• Link(s) to the full text (which may require a subscription or an access fee)
**Cochrane Library – www.thecochranelibrary.com**
Features<br>• Online version (without full-text reviews) accessible at no cost<br>• Contains health-focused Cochrane reviews of impacts (Cochrane Database of Systematic Reviews) and other reviews of impacts (Database of Abstracts of Reviews of Effects and Health Technology

Assessment Database)
- Cochrane Database of Systematic Reviews provides access to scientific abstracts and user-friendly summaries (targeted at lay people). DARE provides links to user-friendly summaries, and the Health Technology Assessment Database provides access to structured scientific abstracts

What is in it?
- Systematic reviews that address questions about the impacts of clinical, health service/system and public/population health interventions, as well as health technology assessments (many of which will contain a systematic review)

How can it be searched?
- Search the entire Cochrane Library or (separately) one of its three most relevant constituent databases
  - Cochrane Database of Systematic Reviews (systematic reviews of impacts produced by members of the Cochrane Collaboration according to defined standards)
  - DARE (systematic reviews of impacts with no restriction on who produced them): Note that the most up-to-date version of this database can be searched separately and that most reviews have a user-friendly summary prepared by the Centre for Reviews and Dissemination - www.crd.york.ac.uk/crdweb/Home.aspx
  - Health Technology Assessment Database - (health technology assessments, which may contain a systematic review): Note that the most up-to-date version of this database can be searched separately and that most reviews have a summary of the HTA's objective prepared by the Centre for Reviews and Dissemination and a link to the full text (which typically does not require a subscription or access fee) - www.crd.york.ac.uk/crdweb/Home.aspx

PubMed/MEDLINE – www.pubmed.gov

Features
- Accessible online at no cost
- Contains many types of health-focused studies, not just systematic reviews. A hedge is available to find systematic reviews (including Cochrane reviews)
- Contains only peer-reviewed articles (i.e. no grey literature)
- Provides links to scientific abstracts only

What is in it?
- Both studies and systematic reviews that address any type of question that may be addressed in the biomedical, clinical, health service/system and public/population health literature

How can it be searched?
- Combine content terms AND terms that will yield systematic reviews, with the terms selected here designed to balance the sensitivity and specificity of a search (emphasising specificity over sensitivity) [17]
  - Cochrane Database Syst Rev [TA] OR search[Title/Abstract] OR meta-analysis[Publication Type] OR MEDLINE[Title/Abstract] OR (systematic[Title/Abstract] AND review[Title/Abstract])
- Possibly also combine with terms that will identify systematic reviews and studies focused on particular jurisdictions or regions (e.g. low- and middle-income countries) – See Appendix 2

What resources are provided for search results?
- A scientific abstract (if available)

Link(s) to the full text (which may require a subscription or an access fee)

Notes<br>• There are versions of MEDLINE that require a subscription (e.g. OVID/MEDLINE)<br>• PubMed contains many types of health-focused studies, not just studies of impacts, and hedges are available for many types of studies

Databases that require subscription access and ideally the support of a librarian

Database	Comments
CINAHL	***What is in it?***<br>• Both systematic reviews and studies that address any type of question (i.e. not just reviews and studies of impacts) that may be covered in the nursing and allied health literature<br>***How can it be searched?***<br>• Combine content terms AND terms that will yield systematic reviews, with the terms selected here designed to optimise the sensitivity and specificity of a search [1]<br>• Confidence intervals (in MH Exact Subject Heading) OR 'dt' (in Word in Major Subject Heading) OR Systematic review (in PT Publication Type) (in CINAHL provided by EBSCO)<br>• Possibly also combine with terms that will identify systematic reviews and studies focused on particular jurisdictions or regions (e.g. low- and middle-income countries)<br>***What resources are provided for search results?***<br>• A scientific abstract (when available)
EMBASE	***What is in it?***<br>• Both systematic reviews and studies that address any type of questions that may be covered in the biomedical and clinical literature<br>***How can it be searched?***<br>• Combine content terms AND terms that will yield systematic reviews, with the terms selected here designed to optimise the sensitivity and specificity of a search [2]<br>• Meta-analys:.mp. OR search:.tw. OR review.pt. (in EMBASE provided by Ovid)<br>• Possibly combine also with terms that will identify systematic reviews and studies focused on particular jurisdictions or regions (e.g. low- and middle-income countries)<br>***What resources are provided for search results?***<br>• A scientific abstract (when available)

PsycINFO	***What is in it?***<br>• Both systematic reviews and studies that address any type of question that may be covered in the psychology literature<br>***How can it be searched?***<br>• Combine content terms AND terms that will yield systematic reviews, with the terms selected here designed to optimise the sensitivity and specificity of a search [3]<br>– Control:.tw. OR effectiveness.tw. OR risk:.tw. (in PsycINFO provided by Ovid)<br>• Possibly combine also with terms that will identify systematic reviews and studies focused on particular jurisdictions or regions (e.g. low- and middle-income countries)<br>***What resources are provided for search results?***<br>• A scientific abstract (when available)
Other databases for which optimal methodology filters for systematic reviews have not yet been developed	***Region-specific interfaces to several of the above-mentioned databases***<br>• Virtual Health Library (Latin America and Caribbean Region)<br>***Regional databases***<br>• African Index Medicus<br>• African Journals Online<br>• Index Medicus for the WHO Eastern Mediterranean Region<br>• Index Medicus for South-East Asian Region<br>• LILACS (Latin America and Caribbean Region)<br>• Western Pacific Region Index Medicus<br>***Global databases with specific disciplinary areas of focus***<br>• EconLit (Economics)<br>• International Bibliography of the Social Sciences (Social sciences)<br>• International Political Science Abstracts (Political science)<br>• ISI Web of Science (Arts and humanities, sciences, and social sciences – citation indices)<br>• PAIS (Public Affairs Information Service) International (Public affairs)<br>• Sociological Abstracts (Sociology)<br>• Wilson Business Abstracts (Management)<br>• Worldwide Political Science Abstracts (Political science)<br>***Disease/condition databases***<br>• TropIKA (Tropical diseases)

7.2 Access to full-text articles and reports (from STP 7)

Mechanism	Comments
**HINARI**	***Who is eligible to use it?***<br>• Institutions in selected low- and middle-income countries have either free access or low-cost access. To check if an institution is already registered or if an institution is located in a country that is el igible for free or low-cost access, go to: HINARI<br>***How can it be accessed?***<br>• An institution must register and all staff are then given unlimited access<br>• Alternatively if a computer is recognised as being based in an eligible country, users may access Highwire Free Access for Developing Countries (which includes HINARI and other selected resources) –<br>***What resources are provided for research results?***<br>• A scientific abstract and full-text article for all included journals
**Cochrane Library**	***Who is eligible to use it?***<br>• Institutions in selected countries have free access – to check if a country (or region) is covered by a programme for low-income countries or by a subscription, go to: Cochrane Library<br>***How can it be accessed?***<br>• Country-or region-specific access details are available at the same site<br>***What resources are provided for research results?***<br>• A scientific abstract, lay summary, and full-text review for all Cochrane reviews, as well a summary of some form for the three most relevant constituent databases described in Table 7.1<br>***Note*** : The Cochrane Library can also be accessed through HINARI
**Journals**	***Who is eligible to use it?***<br>• Anyone<br>***How can it be accessed?***<br>• Websites of open-access journal publishers<br>– BioMed Central (journals beginning with BMC and select others)<br>– OpenJournals Publishing (many journals beginning with 'South African' and select others)<br>– Public Library of Sciences (journals beginning with PLoS)<br>– SciELO (Scientific Electronic Library Online) (many journals from Latin America and the Caribbean)<br>• Directories of open-access and/or free journals<br>– Director of Open Access Journals<br>– Free Medical Journals<br>– Open J-Gate<br>• Repositories through which journal publishers make available articles (often after a defined time period)<br>– PubMed Central<br>– Bioline International (journals from Brazil, Cuba, India, Indonesia, Kenya, South Africa, Uganda, Zimbabwe)<br>***What resources are provided for research results?***<br>• A scientific abstract and full-text article for all included journals

7.3 Sources of national data

To be compiled by the team

NOTE: you may want to use the SUPPORT Tool for local evidence (11) as a guide for identifying and organising sources.

8. Network of experts in Uganda

- To be compiled by the team.

9. Network of experts outside of Uganda

Name	Email	Comments
**Delivery arrangements**		
Bero, Lisa	berol@pharmacy.ucsf.edu	Pharmaceutical policies
Chopra, Mickey	Mickey.Chopra@mrc.ac.za	Human resources
Evans, Tim	evanst@who.int	Human resources, social determinants of health
Garner, Paul	pgarner@liverpool.ac.uk	Malaria, TB, infectious diseases
Gruen, Russ	R.Gruen@alfred.org.au	
Haines, Andy	Andy.Haines@lshtm.ac.uk	
Henry, David	david.henry@ices.on.ca	Pharmaceutical policies
Hill, Sue	HillS@who.int	Pharmaceutical policies
Kelley, Mike	Mike.Kelly@nice.org.uk	Social determinants of health
Lewin, Simon	simon.lewin@nokc.no	Lay health workers
Macintyre, Sally	sally@msoc.mrc.gla.ac.uk	Social determinants of health
Mathews, Cathy	cathy.mathews@mrc.ac.za	Sexually transmitted diseases
Mays, Nick	Nicholas.Mays@lshtm.ac.uk	
Metin Gulmezoglu	gulmezoglum@who.int	MCH
Neilson, Jim	jneilson@liverpool.ac.uk	MCH
Ross-Degnan, Dennis	drossdeg@hms.harvard.edu	Pharmaceutical policies
Sheppard, Sasha	Sasha.Shepperd@dphpc.ox.ac.uk	Location of services
Tharyan, Prathap	prathap@cmcvellore.ac.in	Mental health
Volmink, Jimmy	jvolmink@sun.ac.za	TB, infectious diseases
Zwarenstein, Merrick	merrick.zwarenstein@ices.on.ca	Collaborative care arrangements
**Financial arrangements**		
Bennett, Sara	saracbennett@gmail.com	
Evans, David	evansd@who.int	
Legarde, Mylene	Mylene.Lagarde@lshtm.ac.uk	
Mays, Nick	Nicholas.Mays@lshtm.ac.uk	
McIntyre, Di	Diane.McIntyre@uct.ac.za	
Mills, Anne	Anne.Mills@lshtm.ac.uk	
Palmer, Natasha	Natasha.Palmer@lshtm.ac.uk	
Ranson, Michael Kent	ransonm@who.int	
Savigny, Don de	d.desavigny@unibas.ch	
Tan Torres Edejer, Tessa	tantorrest@who.int	
Vale, Luke	l.vale@abdn.ac.uk	
**Governance arrangements**		
Bero, Lisa	berol@pharmacy.ucsf.edu	Conflicts of interest
Gilson, Lucy	Lucy.Gilson@uct.ac.za	
Hill, Sophie	sophie.hill@latrobe.edu.au	Consumer involvement
Koechlin, Lucy	lucy.koechlin@baselgovernance.org	
Mays, Nick	Nicholas.Mays@lshtm.ac.uk	
McKee, Martin	martin.mckee@lshtm.ac.uk	
Oliver, Sandy	S.Oliver@ioe.ac.uk	Consumer involvement
**Implementation**		
Althabe, Fernando	falthabe@gmail.com	MCH, Health professionals
Eccles, Martin	Martin.Eccles@ncl.ac.uk	Health professionals
Garner, Paul	pgarner@liverpool.ac.uk	Malaria, TB, infectious diseases

Name	Email	Comments
Glenton, Claire	Claire.Glenton@sintef.no	Patients, health care recipients
Grimshaw, Jeremy	jgrimshaw@ohri.ca	Health professionals
Gruen, Russ	R.Gruen@alfred.org.au	
Haines, Andy	Andy.Haines@lshtm.ac.uk	
Hill, Sophie	sophie.hill@latrobe.edu.au	Patients, health care recipients
Hofmeyr, Justus	gjh@global.co.za	MCH
Langer, Ana	alanger@engenderhealth.org	MCH
Lewin, Simon	simon.lewin@nokc.no	Patients, health care recipients
Mathews, Cathy	cathy.mathews@mrc.ac.za	Sexually transmitted diseases
Metin Gulmezoglu	gulmezoglum@who.int	
Neilson, Jim	jneilson@liverpool.ac.uk	MCH
Ross-Degnan, Dennis	drossdeg@hms.harvard.edu	Health professionals
Tharyan, Prathap	prathap@cmcvellore.ac.in	Mental health
Volmink, Jimmy	jvolmink@sun.ac.za	TB, infectious diseases
Zwarenstein, Merrick	merrick.zwarenstein@ices.on.ca	Health professionals

10. Examples

- To be added as they are accumulated from the pilot.
