# Supplementary figures and images for: Feasibility of a rapid response mechanism to meet policymakers' urgent needs for research evidence about health systems in a low income country: a case study
Source: Implement Sci. 2014 Sep 10;9:114. doi: 10.1186/s13012-014-0114-z (PMC4172950; doi:10.1186/s13012-014-0114-z)

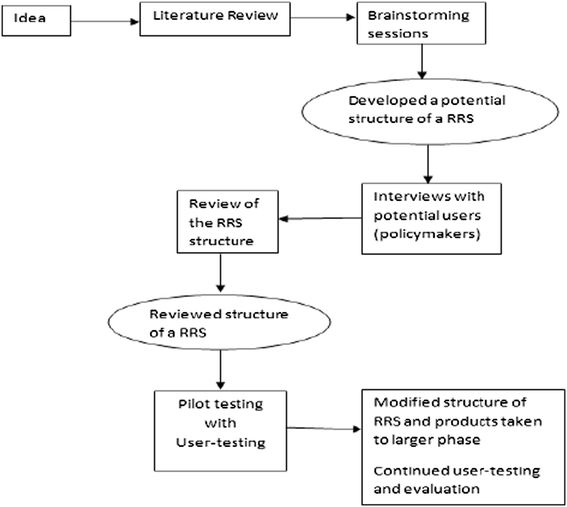

Supplement: Supplementary file 2 — Authors’ original file for figure 1 [file 13012_2014_114_MOESM2_ESM.gif]

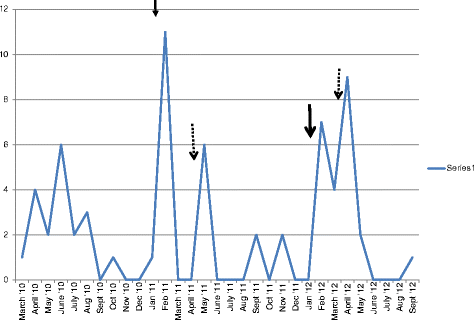

Supplement: Supplementary file 3 — Authors’ original file for figure 2 [file 13012_2014_114_MOESM3_ESM.gif]
