# Supplementary material for: Feasibility of a rapid response mechanism to meet policymakers' urgent needs for research evidence about health systems in a low income country: a case study
Source: Implement Sci. 2014 Sep 10;9:114. doi: 10.1186/s13012-014-0114-z (PMC4172950; doi:10.1186/s13012-014-0114-z)
Supplement: Supplementary file 4 — Authors’ original file for figure 3 [file 13012_2014_114_MOESM4_ESM.docx]

**Table 2: Organization of affiliation of rapid response service users**

| **Organization of affiliation of Policymakers** | **Number of Policymakers** |
| --- | --- |
| Ministry of Health | 23 |
| Bi/Multi-lateral Organizations | 4 |
| Government (Not Ministry of \health) | 2 |
| Non-Governmental Organizations | 1 |
| Districts | 0 |
| Total | 30 |
