# Supplementary material for: Feasibility of a rapid response mechanism to meet policymakers' urgent needs for research evidence about health systems in a low income country: a case study
Source: Implement Sci. 2014 Sep 10;9:114. doi: 10.1186/s13012-014-0114-z (PMC4172950; doi:10.1186/s13012-014-0114-z)
Supplement: Supplementary file 5 — Authors’ original file for figure 4 [file 13012_2014_114_MOESM5_ESM.docx]

**Table 3: Topics or areas for research evidence needs posed to the rapid response service**

| **Type of question** | **Frequency** |
| --- | --- |
| Governance | 17 |
| Organization | 13 |
| Health Technology Assessments | 11 |
| Implementation Strategies | 11 |
| Financial Arrangements | 9 |
| Other (E.g. Public health) | 4 |
| Total | 65 |
