# Supplementary material for: Feasibility of a rapid response mechanism to meet policymakers' urgent needs for research evidence about health systems in a low income country: a case study
Source: Implement Sci. 2014 Sep 10;9:114. doi: 10.1186/s13012-014-0114-z (PMC4172950; doi:10.1186/s13012-014-0114-z)
Supplement: Supplementary file 6 — Authors’ original file for figure 5 [file 13012_2014_114_MOESM6_ESM.docx]

**Figure 5: Histogram showing the frequency of receipt of questions by the Rapid Response Service**
