# Supplementary material for: Feasibility of a rapid response mechanism to meet policymakers' urgent needs for research evidence about health systems in a low income country: a case study
Source: Implement Sci. 2014 Sep 10;9:114. doi: 10.1186/s13012-014-0114-z (PMC4172950; doi:10.1186/s13012-014-0114-z)
Supplement: Supplementary file 7 — Authors’ original file for figure 6 [file 13012_2014_114_MOESM7_ESM.docx]

**Table 4: Table showing confidence in respondents’ own answers at the time of asking Rapid Response Service help**

| Confidence in answers before rapid response | Frequency | Percent |
| --- | --- | --- |
| Very confident | 1 | 1.5 |
| Confident | 6 | 9.2 |
| Neither confident nor ‘unconfident’ | 30 | 46.2 |
| ‘Unconfident’ | 19 | 29.2 |
| Very ‘unconfident’ | 3 | 4.6 |
| No idea | 3 | 4.6 |
| No response | 3 | 4.6 |
| Total | 65 | 100.0 |
