# Supplementary material for: Feasibility of a rapid response mechanism to meet policymakers' urgent needs for research evidence about health systems in a low income country: a case study
Source: Implement Sci. 2014 Sep 10;9:114. doi: 10.1186/s13012-014-0114-z (PMC4172950; doi:10.1186/s13012-014-0114-z)
Supplement: Supplementary file 8 — Authors’ original file for figure 7 [file 13012_2014_114_MOESM8_ESM.docx]

**Table 5: Table showing confidence in the respondents’ course of action on given decision before Rapid Response Service help**

| Confidence in course of action before rapid response | Frequency | Percent |
| --- | --- | --- |
| Very confident | 1 | 1.5 |
| Confident | 11 | 16.9 |
| Neither confident nor ‘unconfident’ | 26 | 40.0 |
| ‘Unconfident’ | 18 | 27.7 |
| Very ‘unconfident’ | 5 | 7.7 |
| No idea | 2 | 3.1 |
| No response | 2 | 3.1 |
| Total | 65 | 100.0 |
