# Supplementary material for: Feasibility of a rapid response mechanism to meet policymakers' urgent needs for research evidence about health systems in a low income country: a case study
Source: Implement Sci. 2014 Sep 10;9:114. doi: 10.1186/s13012-014-0114-z (PMC4172950; doi:10.1186/s13012-014-0114-z)
Supplement: Supplementary file 9 — Authors’ original file for figure 8 [file 13012_2014_114_MOESM9_ESM.docx]

**Table 6: Table showing respondents’ change in answers after using the rapid response brief**

| Change in answer after rapid response brief | Frequency | Percent |
| --- | --- | --- |
| Yes | 43 | 66.2 |
| No | 14 | 21.5 |
| No response | 8 | 12.3 |
| Total | 65 | 100.0 |
