# Supplementary material for: Feasibility of a rapid response mechanism to meet policymakers' urgent needs for research evidence about health systems in a low income country: a case study
Source: Implement Sci. 2014 Sep 10;9:114. doi: 10.1186/s13012-014-0114-z (PMC4172950; doi:10.1186/s13012-014-0114-z)
Supplement: Supplementary file 10 — Authors’ original file for figure 9 [file 13012_2014_114_MOESM10_ESM.docx]

**Table 7: Table showing change in respondents’ course of action following rapid response brief**

| Change in course of action following rapid response brief | Frequency | Percent |
| --- | --- | --- |
| Yes | 30 | 46.2 |
| No | 25 | 38.5 |
| No response | 10 | 15.4 |
| Total | 65 | 100.0 |
